# Detection of Hidden Hostile/Terrorist Groups in Harsh Territories by Using Animals as Mobile Biological Sensors

**DOI:** 10.3390/s8074365

**Published:** 2008-07-25

**Authors:** Yasar Guneri Sahin, Tuncay Ercan

**Affiliations:** 1 Izmir University of Economics, Department of Software Engineering, Sakarya Cad. No:156 Balcova/Izmir – Turkey; 2 Yasar University, Department of Computer Engineering, Kazim Dirik Mah. 364.Sok, Bornova/Izmir Turkey; E-mail: tuncay.ercan@yasar.edu.tr

**Keywords:** Mobile biological sensors, location detection, mobile sensor networks, military operations

## Abstract

Terrorism is the greatest threat to national security and cannot be defeated by conventional military force alone. In critical areas such as Iraq, Afghanistan and Turkey, regular forces cannot reach these hostile/terrorist groups, the instigators of terrorism. These groups have a clear understanding of the relative ineffectiveness of counter-guerrilla operations and rely on guerrilla warfare to avoid major combat as their primary means of continuing the conflict with the governmental structures. In Internal Security Operations, detection of terrorist and hostile groups in their hiding places such as caves, lairs, etc. can only be achieved by professionally trained people such as Special Forces or intelligence units with the necessary experience and tools suitable for collecting accurate information in these often harsh, rugged and mountainous countries. To assist these forces, commercial micro-sensors with wireless interfaces could be utilized to study and monitor a variety of phenomena and environments from a certain distance for military purposes. In order to locate hidden terrorist groups and enable more effective use of conventional military resources, this paper proposes an active remote sensing model implanted into animals capable of living in these environments. By using these mobile sensor devices, improving communications for data transfer from the source, and developing better ways to monitor and detect threats, terrorist ability to carry out attacks can be severely disrupted.

## 1. Introduction

Terrorism is defined as: “An illegal preconceived use of physical or psychic violence (or threat of it) for further political goals aimed at civilians or non-combatants etc., to change current policies, their methods and structure” [1, 2]. More, The United States Department of Defense defines terrorism as “the calculated use of unlawful violence or threat of unlawful violence to inculcate fear; intended to coerce or to intimidate governments or societies in the pursuit of goals that are generally political, religious, or ideological” [3].

Afghanistan, Iraq, Turkey, and other countries with terrorist problems have large areas of uninhabited land, often consisting of rugged terrain with national borders, roads, rivers and industrial facilities that need monitoring to prevent terrorists and hostile groups from entering the country, damaging infrastructure, mining roads, attacking government units and moving freely [4, 5, 6]. The effective control of internal security operations especially in the harsh terrain is extremely important for national security, however difficult ground conditions make regular combat and engagement almost impossible.

Many terrorist groups or even individuals acting alone may hide in caves or other places that are are difficult to observe in the daytime, and attack at night. It is too difficult to plan a day-time attack against an enemy to whom borders mean nothing. In such a situation, the best strategy is to use intelligence, finance and psychological warfare to find and destroy terrorists' hidden bases. Military forces must prevent these hostile groups from taking over a part of the country and establishing a local control of remote areas, which can become a base for launching future terrorist operations. Therefore, they must counter these particular guerrilla tactics with special doctrine and training. The terrorist groups are small forces equipped with light weapons and simple command, control, and information systems. They easily blend into the civilian population. At present regular military forces lack the investigative skills to track down these people and in some circumstances have few information sources in the civilian population to enable discrimination between friend and foe. Thus, there is a clear need for the military and police units to deal with such challenges and these entities. These entities need to adapt new and emerging technologies to locate these inimical groups using various levels of different remote sensing devices, as well as detecting chemical explosives. They especially need more rapid, simpler, more cost effective and more accurate tools for detecting and identifying a wide spectrum of usable human specific information that could be the basis for a counter attack on terrorists.

Networked sensor systems are seen as an important technology that will reach the full extent of deployment in the next few years for a number of applications, not only for national security [7, 8]. A sensor network is an infrastructure comprised of sensing, measuring, computing, and communication elements that gives the users the ability to observe and react to events and phenomena in a specified environment.

Habitat and environmental monitoring represent a class of sensor network applications with many potential benefits for scientific and social issues. However, the problem of using sensor equipment in mountainous regions means that the sensor system is difficult to set up. Using animals as mobile sensors resolves the problem of the placement of static sensors in inaccessible terrain. The intimate relation between domestic animals and human beings in a certain region allows each sensor implanted or tagged into these animals to collect detailed information and provide localized measurements to detect the hidden groups in the field. Previously stated methods which are similar in regard to animals tracking with sensors and early fire detection may easily be applied to these situations. While the sensor users track animals to gather information on their usual habits, such as hunting or mating, they can also gather data which detects human presence and activity [9].

The challenge is to set up a wireless sensor network in geographically harsh and rugged areas to enable regular military units to use animals as mobile sensor agents. These agents will be indispensable in gathering necessary data to combat terrorist groups hidden in this environment. The design and implementation of the proposed sensor services is evaluated in this paper. Furthermore, this paper reviews mobile sensor network architectures and provides background information for some animals that can be used as mobile sensors in the harsh terrain, including relevant definitions and a technical summary of space-based remote-sensing.

## 2. Related Works

Animal behavior research using different kind of sensors has been carried out for years and a great deal of scientific research has been conducted with regard to the existence and habitat of the marine and land animals. In these studies, animals with sensors attached, have been tracked and meaningful data that can be used to classify many different behaviors has been collected [10-13]. Hence, this technique can provide beneficial data that can be used for many different purposes. A new frontier was opened with Khan's idea that is the most efficient and cost effective biosensors are already distributed globally but generally ignored: they are called animals [14].

The detection of hostile groups and terrorist locations, border control and prevention of illegal immigration have been investigated for many years and much previous work is available [15-31]. In particular, exploration and innovation in space and satellite systems have caused scientists to focus on this technology to track environmental and other features. Capturing pictures using these satellites also provides meaningful data for understanding the behavior of the object being tracked. The commercial availability of satellite pictures presents a great benefit to national security and military operations. For example, the military has advantages to access this high-resolution satellite imagery, with which the movement of terrorist groups in the field, their build-up and the lay-out of their camp sites, facilities and even the locations of individuals could be made available to the Government Security Organizations. It is obvious that there should be a balance between maintaining the same capability to these terrorist groups and protecting the national security.

Space-based remotely sensed imagery became commercially available worldwide in 1972 with the advent of the United States (U.S.)'s Earth Resources Technology Satellite (ERTS). Until that time, such information was within the province of military and intelligence communities of the major world powers. With the launch of the French “Systeme Probatoire d'Observation de la Terre (SPOT) I” satellite in 1986, remotely sensed imagery with ten meter resolution became commercially available. Today, Commercial Space Imaging Companies sell images with better than one meter resolution on the international market. Remote sensing from space can be as simple as taking photographs of the Earth from space using an optical camera and photographic film. Sensors on board a satellite in either geosynchronous orbit (GEO), or an inclined or polar low-Earth orbit (LEO), perform remote sensing from space [32]. In addition to the numerous civilian applications, this technology is also used for military reconnaissance and verification of compliance with arms control treaties, contributing to world security [33]. The command and control system of the terrorist groups leave Radio Frequency (RF) signatures easily targeted by the Military Electronic Units. Even though terrorist groups need a centralized command and control of their activities, they can sometimes operate on very slow communications, or even hand written notes carried by human messengers from the civilian population, making activity much more difficult to detect.

One of the most important discoveries in the detection of these terrorist groups is the use of unmanned aircraft, such as Scout, Searcher, Heron, Hunter, Pioneer, Harpy, Ranger, I-See, I-View, which, because of special features, make detect of inimical groups much easier, using evaluated data (images and video) sent from 3000 m or higher [34]. These planes with night vision are able to observe the movements of groups at night. However, these aforementioned methods are based on images of previously found detection, which means that the group must have been already located. This study proposes a system for locating hidden groups in places where previously no valid signals have been detected.

Building on the previous uses of sensors (in environmental detection) our proposal constitutes a practical and important addition to mobile sensor usage in the form of tagging or attaching sensors to domestic animals: the detection and location of terrorist groups hidden in caves and harsh environments. While the system proposed does not claim to discover every possible human target, it represents an easily employed additional reconnaissance tool for the benefit of Special Forces.

## 3. Motivation

There are two drawbacks to current surveillance methods, the first of which is training. The terrorists and hostile groups act like peaceful civilians, making identification difficult. While national intelligence units try to improve gathering of human intelligence data with combination of various intelligence sources, surveillance and reconnaissance in the tactical area, without training in exploiting these alternative sources of intelligence, no real progress can be made.

The second drawback is the effect of international obligations. Satellite remote sensing is the most useful resource for supporting the initial identification and relative location of targets, transportation routes used by criminals, terrorists, and enhances the offensive military capability. Images obtained from satellites can be used to generate real time simulations of targets for the specialized training of personnel such as Special Forces, and prepare them for operations in unfamiliar terrain. These images can also help anticipate problems that could arise during the attacks and disclose of tactical flaws in the operation plans. However, foreign policy and international obligations cause significant limitations for resolution and system throughput due to national security. For example, the operational limitations only permit the collection of images with a one meter resolution and dissemination no earlier than 24 hours after data collection, although current systems may, in fact, be capable of producing images with a half-meter resolution in a matter of hours. This puts commercial operators in the unique position of possessing highly sensitive remote sensing data unavailable to the rest of the world [35].

Sensor networks can improve detection and tracking performance with multiple observations. Wireless sensor networks are mainly mesh network systems with multi-hop radio connectivity among or between the sensor nodes used in the environment. Military theater systems of this type of networks provide applications of environmental monitoring and national security systems. Existing and potential applications of sensor networks include; military sensing, physical security, military applications, monitoring inimical and friendly forces, military-theater or battlefield for reconnaissance and surveillance, weather sensing, environment monitoring, and national border monitoring [36, 37].

With advances in digital technology, it is becoming practical to provide sensors with a wireless connection that enables remote access to the inputs and outputs of devices. Through a variety of location technologies, data from both fixed and mobile sensing devices can be used to report geographical location. The use of wireless and mobile sensor networks will limit the need for military personnel involvement in dangerous reconnaissance missions [38]. The computing and networking capabilities allow sensor networks to be reprogrammed or re-tasked after each deployment in the field. Nodes have the ability to adapt the operation by time in response to changes in the environment [39]. It is impossible to predict the real time conditions in which it would be necessary to limit data collection and distribution according to a certain course of action against the targets. Any transfer delay between the sensor environment and the authorized users may create national security problems, which may even lead to military causalities.

In the short term, the communication links and the satellite ground stations are potentially the most vulnerable components during crises or wartime, when active and passive sensing should be impeded by the owners of satellites. In addition, electronic jamming can be used against the ground station to disrupt the uplink and downlink features. Alternatively, the remote sensing system could be crippled by launching a military assault on the ground receiving station [40].

**Figure 1.**
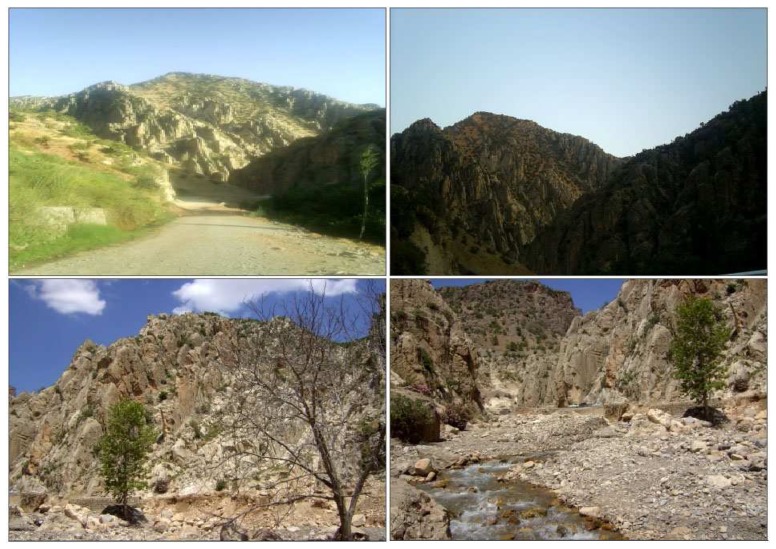
Possible application areas of the system.

In contrast to these disadvantages of satellite sensors from the point of national and military security, land based sensor networks are much less vulnerable to attacks, and can operate in an unattended fashion in dispersed and/or remote geographic locations: Nodes may be deployed in harsh, hostile, or widely scattered environments, which give rise to challenging management mechanisms. Figure 1 shows the probable environments where the systems can be used, where it would be more suitable to exploit the local tracking systems using the GPS features because of the constraints in satellite use and the limiting factors of the territory.

## 4. Animals as Mobile Biological Sensors for Harsh Terrains

Communications technologies enable rapid and accurate identification, location tracking, and condition monitoring of high-value assets. The applications of mobile biological sensors include identification and monitoring of humans, pets, fish, poultry and other livestock through patented implantable microchips, the location tracking and message monitoring of vehicles and aircraft in remote locations through systems with GPS and/or geosynchronous satellite communications; and monitoring of asset conditions such as temperature and movement, through advanced miniature sensors. The miniaturization of electronic components and sensors (MEMs usage and technology) combined with advances in satellite technology have led to dramatic improvements in the tags, great increasing the ability to pinpoint an animal's geographic location, and researchers are continuing to add new capabilities.

### 4.1. Proposed System Structure

**Figure 2.**
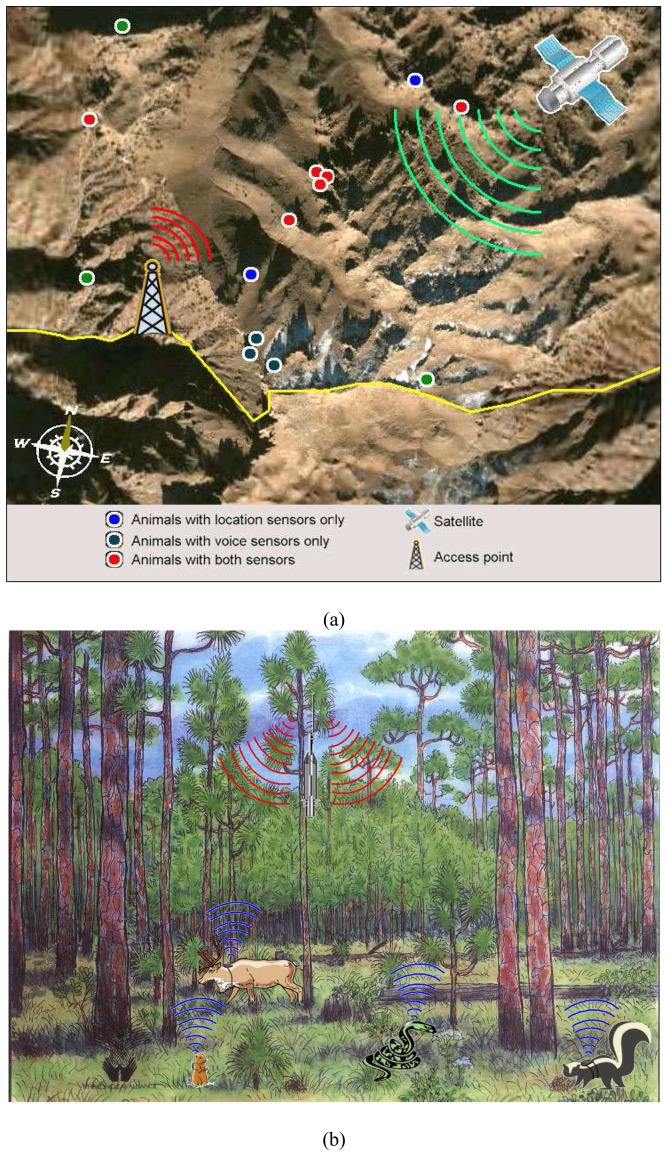
System operating structure.

The system has been based on two fundamental methods of measurement. The first involves attaching sensors which use GPS data to detect potential hiding place. Similarly, the second also uses tiny sensors that are virtually invisible, to detect human voices and locate their source using GPS signals. Figure 2 shows the general architecture and animal tracking methods: a) for mountain region b) for forests.

#### 4.1.1. Possible hidden places detection method (HPD)

Detection of hidden places using animals consists of three main components: an animal (preferably moving frequently within a large territory) equipped with a sensor which has GPS capability, satellites to obtain GPS data and location detection software.

**Figure 3.**
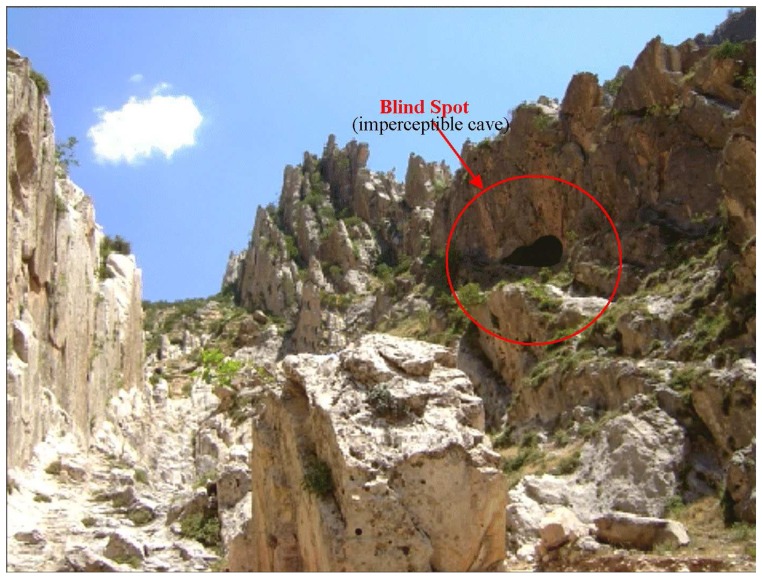
Possibly imperceptible cave.

**Figure 4.**
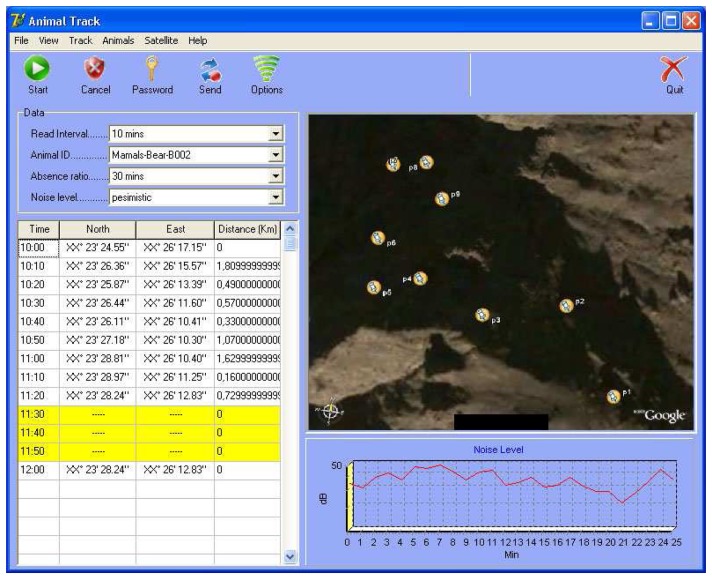
Detection software screenshot.

The system works in a simple way; Animals are continuously observed and location detection data is regularly obtained (every 1-20 minute). Using this data, the probable route of movement of the animal, hunting area and places to shelter can be projected. The location information acquired from the GPS signals also provides data for the length of time the animal cannot be tracked. The invisibility intervals are considered to be caused by loss of GPS signals, and these blind spots are considered as caves or other potential terrorist hiding place. Figure 3 shows a cave that can be used as a hidden place. As shown in the figure, there will be no signal if the animal enters a cave, but the signal will reappear when it emerges, thus indicating a probable hiding place in this location. Figure 4 shows a screen shot of the software program developed to find a probable hidden place and the audio tracking system.

#### 4.1.2. Sample HPD scenario

The followings are the steps of the location detection process in the software:

1. Firstly, the reading interval is adjusted according to the number of animals used. This interval shows data flow frequency for each animal. In the sample application, it has been taken as 10 minutes.
2. One of the animals with an injected sensor in advance is selected, using Animal ID. In this example, B-002, “bear” has been selected.
3. The “Absence Ratio” is determined. This value changes with the animal type. Fast, frequently moving animals should have smaller reading interval and “absence ratio”. In this example, absence ratio for the “bear” is 30 minutes.
4. “Noise level” is a variable used to recognize the human voice. If the animal has audio sensors, these are used to decompose between the different volumes of human voice. “Pessimistic” selection in this field is for the detection of very low level human voices in the critical territories. However, it was not used in this example, since signal tracking method was used for location detection.
5. The location grid in the software shows the tracking periods and coordinates of the selected animal. A different color indicates no signal for a 30 minutes period taken as the absence ratio. If the signal is picked up again, the distance information in the table is checked. A value of between 0 and 10 meters (these ratios can be defined as parameters) clearly demonstrates that the animal stayed in a place from where no signal could be recorded in the period of not less than the absence ratio.
6. Security forces determine and check the location of first signal loss (considered as entrance point for hiding place) and where the signals re-establish themselves.

Some predictions can be made depending on the animal used, a bear in this example. Because of the size of the bear the cavity can be considered correspondingly large. It is important to select parameters according to the size and features of animals, as well as regional features, and therefore cooperation with a zoologist with the required knowledge is essential.

#### 4.1.3. Human voice detection method (HVD)

Human voice detection system by using animals has already been in frequent use and is only the decomposition of the human voice by using the sensors tacked into animals. The system includes four main components: the animals which have implanted sensors with GPS features, voice recording and tracking (small and low profile mammals preferred), GPS satellites, base stations/signal towers for signal detection and the software for location and detection in turn.

It is fairly simple to recognize a terrorist by the human voice detection method; Animals used are continuously observed, evidence to assess the possible presence of any human in the environment are obtained by the location information taken from the animals through the signal towers every 1-2 minutes. By processing the location and voice information, the possibility of human voice presence can be assessed, and, if strong sensors are used, conversations can be overheard. Figure 4 below shows screenshots of software specially prepared for such a system. There is an “Audio Spectrometer” to check the density and variation of voice waves. The location detection method of probable hidden places can also be used in cases where the signals have been lost. However, in the system there may be signal losses due to the small animals used hiding in small places which humans could not use. In this situation, evaluating these places as to whether they could be potential terrorist shelters is essential for security.

#### 4.1.4. Sample HVD

The steps of the process of detecting voice by the software used for recognizing human voice and location detection are as follows:

1. Firstly, the intervals at which reading is taken are determined according to the infrastructure and the number of animals used in the system. This interval shows how often any particular type of animal will provide data. In this example, the interval has been determined as 1 minute.
2. Any sensor carrier animal is chosen using Animal ID (previously assigned to the animals). In this example, a vole with the ID V-001 was selected.
3. The next stage is determining the absence ratio, which in this example is used for checking (data), as the voice signals. This ratio varies according to the animal used; the absence ratio and reading data interval should be smaller for the fast and shuffling animals. It has been taken as 5 minutes for vole in this example.
4. Noise level is a variable which is used in the scanning of human voice. It is used to discriminate the different levels of human voice from sensored animals. “Pessimistic” selection in this field is used for human voices at very low volumes in critical zones, whereas “Optimistic” is a parameter to filter low frequency human voices in probable places. In this example, “pessimistic” has been chosen in order to be used in the critical regions.
5. The movement of the animal with the coordinates and time spent is shown in the location grid available in the software. A different color is used if there is no signal for more than 5 minutes in the absence ratio. The distance is checked when the signal is reachable again, and a reading of 0 to one meter is considered to indicate that the animal remained in a location that did not allow a signal for that duration of time (assuming that it is not less than the absence ratio).
6. The voice sensors implanted into the animal are filtered to determine whether they contain human voices or not. Any human voice signals are transmitted to the software by the signal towers and shown on a spectrometer. These signals can also be listened to by the software user. If any significant and characteristic human voice is recognized, the position of the animal is determined and checked.
7. While this happening, it is possible to identify and confirm the location of the animal disappearance (the point where the signal was lost) and reappearance (the point where the signal resumes), as well as features of the voice being tracked.

Some predictions can be made about the hiding places of the animal used in this sample, a vole. Since its hiding places are likely to be very small, uncertainty arisen as to whether these are suitable places for humans to shelter, and thus there is a need to consult someone familiar with the region. In addition, consultation with zoologists and guides with relevant expertise are necessary to select parameters according to animal and regional features.

### 4.2. Suitable Animals

**Figure 5.**
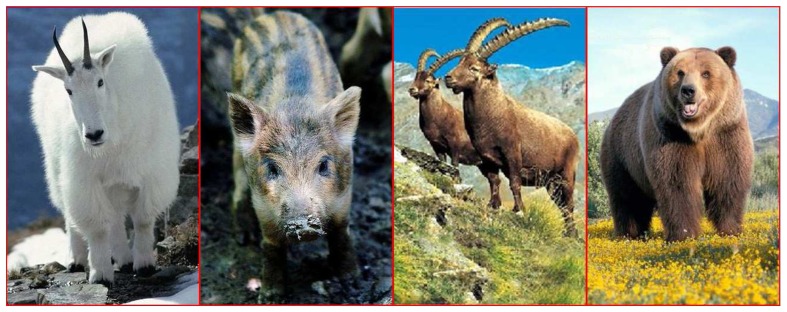
Special mammals for harsh territories.

**Figure 6.**
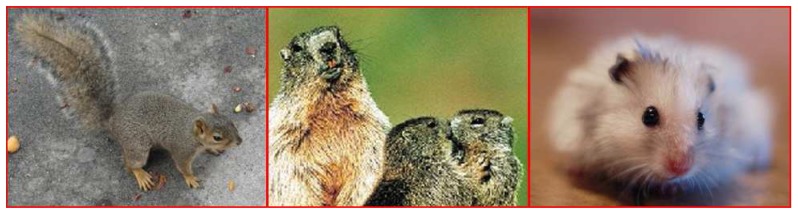
Special rodents for harsh territories.

Animals can easily adapt themselves to life under harsh conditions. Some animals particular to the area (for example goats, hamsters and rabbits in mountainous regions) freely move, hunt and live in environments that are completely inaccessible to humans [42, 42]. Figures 5 and 6 show some of the wild animals that can carry sensors and live in these environments.

In this study, animals have been divided into two different groups for examination according to the systems applied. The first group is the large mammals that can be used for the hard terrain and the detection of probable hiding places of terrorists (HPD). The bear, mountain goat, wild boar and local animals in Figure 5 are examples of these. Large mammals particular to the local area can be equipped with GPS location detection devices and be tracked continuously for location detection.

The second group are the small animals that can be used to detect the voice signals to obtain understandable human voice (HVD). Voles, hamsters, squirrels and the local animals in Figure 6 are examples of these small and hard to detect animals. They move rapidly and hunt in limited areas. When they sense a danger and cannot run away, they keep still and wait for the danger to pass, because of their hiding instinct. They are easily affected by the human moving around in the area. Because sensors implanted into small animals can be used for both detection systems, they can be used to detect both human mobility and communication.

In addition, other animals particular to the region can be specially trained for the proposed systems. In order to make such a selection, it is necessary to coordinate with a zoologist who knows the environment.

### 4.3. Suitable Sensors

**Figure 7.**
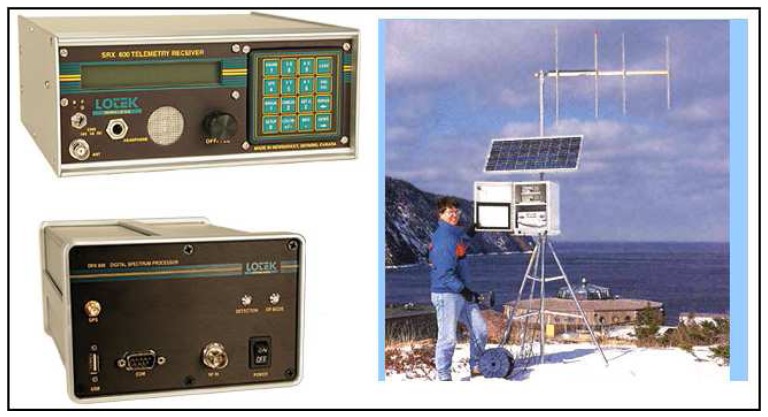
Sample data transceivers for signal towers [43-47].

The electronic components (the sensors tacked into the animals, the signal towers which will provide the data transfer and data collectors) used in these two systems should be selected carefully and suitable for to the conditions of the region. Some examples of data collectors and transmitters which can be used according to the changing conditions in the region are given in Figure 7.

**Figure 8.**
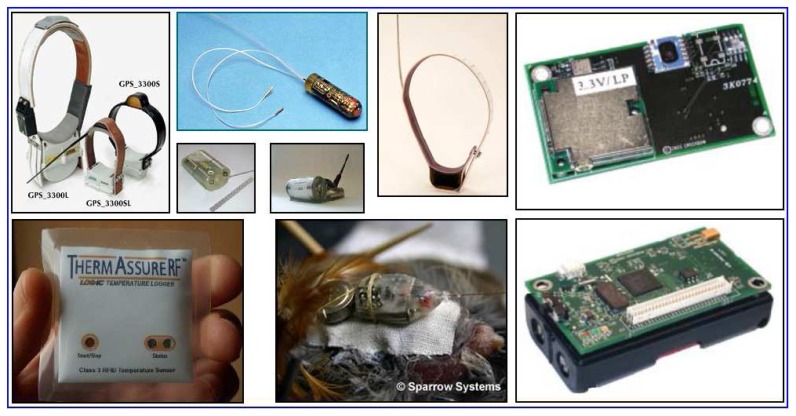
Sample sensors that can be used in the system [9, 43-47].

Some GPS, location and voice detection sensors which can be used on animals are shown in Figure 8. The sensors should be selected according to the features of the region and the characteristics of animal. Selection parameters can have different values according to the size of the animals, the distance from the signal tower and the scale of the region. There are many sensor companies and sensor types in the market, but the important point is to avoid using sensors which put the wild life at risk and damage their habitat.

## 5. Simulation Results and Discussion

Since the testing of the system in real environments are limited and the system features have military value, simulation results and the implications from these results will be presented in this chapter. Map samples taken from Google [48] have been used in the software and simulation studies.

**Figure 9.**
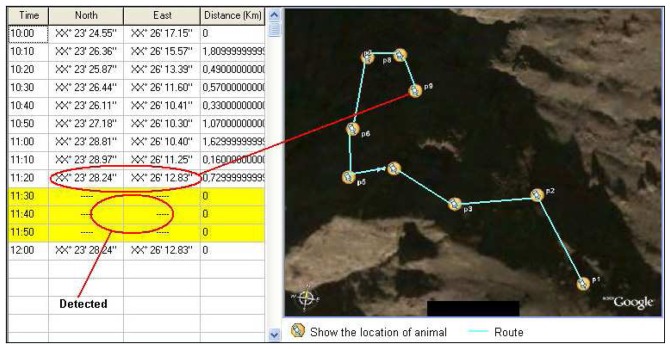
Sample shot from simulation result for HPD.

Figure 9 shows a screenshot including simulation results applied to the HPD method. The system clearly points out the latest spot according to the predetermined simulation results. However, since the distance values are given as “kilometer” for the data obtained in the simulation, there is 1-50 meter diversion, and therefore the hidden place has been calculated as a circular area of 50 meters in diameter rather than a specific point.

**Figure 10.**
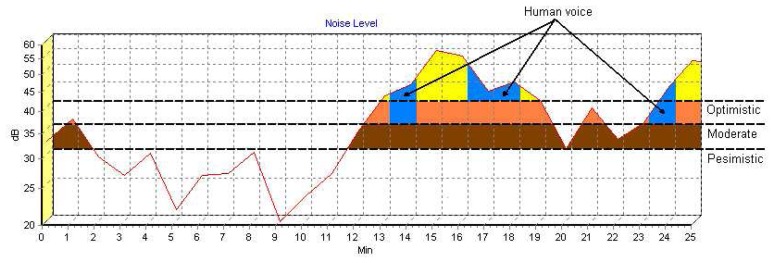
Simulation results for HVD.

Figure 10 shows the graphic including simulation data applied for the HVD method. This graphic, also displays the spectrometer of the recorded voice signals by the animal. The values for all noise levels (pessimistic, moderate, and optimistic) are shown separately. Voice levels for the pessimistic, moderate and optimistic levels are shown by brown, orange and yellow areas respectively. Additionally, blue regions in the figure show the areas of probable human voice detection. It is possible to listen and understand voice strengths of optimistic and above. There are, however, some problems in this sampling, the most important of these is limiting data flow interval. On the other hand, if there is a delay of (even) 2-3 minutes between the recording of the voice data and the decomposition and definition, there is a risk of being unable to pin point the exact location because the detection individuals may move in this time. Consequently data flow intervals should be minimized.

There are advantages and disadvantages of these methods used to detect existence of human activity and the probable hidden places in the targeted region by processing the data acquired from the animals.

### Problems and Disadvantages

1. It is a challenge to catch the animals from the environment and implant sensors into them in order to set up the system.
2. When specially-trained animals are used for this system, it can create problems related to animal rights.
3. There may be an insufficient number of animals particular to the region.
4. Since the power of sensors is generally provided by batteries, lack of battery power or physically damaged devices can disrupt the regular data collection.
5. The cost of equipment used for the decomposition of human voice signals is considerable (although this may not be a constraint where national security is concerned). The natural reactions of animals equipped with sensors should always be examined before. There is also a necessity of using more than one group of animals to detect multiple human groups.
6. As soon as the audio signals have been recorded, it is essential keep a very short data flow interval because it is likely that the target (voice owner) will move.
7. The high cost of and difficulty in installing the data collection tower infrastructure especially HVD method.

### Advantages

1. It is adaptable to the existing location detection systems and it is productive in terms of early detection.
2. Using animals as mobile biological sensors is preferable to using fixed sensors because it allows measurement to be taken at any point in a region.
3. It allows the possibility of obtaining information using fewer of sensors than the fixed systems.
4. It allows the possibility of monitoring areas unreachable by satellites and unmanned aircrafts, using sensor equipped animals moving freely in their habitat.
5. The infrastructure of the system can provide alternative applications other than data collection process. It also enables the possibility of obtaining information about various types of animal.
6. It is easily adaptable to predesigned animal tracking systems.
7. It provides an important support to the prevention of poaching because the sensors allow the immediate detection of death of the animal.

Although the advantages, disadvantages and the aforementioned problems may vary according to different conditions, the advantages are still noteworthy.

## 6. Conclusions

This paper proposes an approach the detection of terrorist groups in their hiding places, in mountainous and forest regions, using animals particular to the region as mobile biological sensors. The paper is based on proposed additions to sensors used in former zoological studies. This provides an extra capability in any related field of zoology. In almost every type of harsh territory, where the satellites and unmanned aircrafts cannot be used despite their advanced equipment, it provides early detection ability against possible terrorist acts and illegal trade across national border lines. Since the system cannot be tested in the real environment at present, it may be affected by the aforementioned problems. In addition, while there may be other (unforeseen) negative effects not considered so far, the system outlined here would seem practical and effective, and worth building up.

The followings issues seem desirable topics for future studies:

1. Studies to classify the animal movements would help to build more effective systems in the future.
2. Improving sensors would make systems run more effectively.
3. Studies using animal psychology to produce animals adapted to the needs of the system would be helpful.
4. Integrating wireless networking infrastructure and satellite tracking systems with new and advanced technologies would make the system more effective.
5. Embedded early reaction techniques could be employed for future studies, not only for detection but also for an effective reaction process.

Consequently, these types of studies may lead to effective measures against terrorism and smuggling, especially in the terms of serious activities which endanger the national and global security. A novel proposal to eliminate this threat described in this study, and it could be an effective tool for future works.
